# Measures of Adiposity and Risk of Stroke in China: A Result from the Kailuan Study

**DOI:** 10.1371/journal.pone.0061665

**Published:** 2013-04-17

**Authors:** Anxin Wang, Jianwei Wu, Yong Zhou, Xiuhua Guo, Yanxia Luo, Shouling Wu, Xingquan Zhao

**Affiliations:** 1 Department of Neurology, Beijing Tiantan Hospital, Capital Medical University, Beijing, China; 2 Department of Epidemiology and Health Statistics, School of Public Health, Capital Medical University, Beijing, China; 3 Department of Cardiology, Kailuan Hospital, Hebei United University, Tangshan, China; University of Cambridge, United Kingdom

## Abstract

**Objective:**

The objective of this study was to explore the association between adiposity and risk of incident stroke among men and women.

**Methods:**

We studied the relationship between adiposity and stroke among 94,744 participants (18–98 years old) in the Kailuan study. During a follow-up of 4 years, 1,547 ischemic or hemorrhagic strokes were recorded. Measurements of adiposity included body mass index (BMI), waist circumference (WC), waist-to-hip ratio (WHpR), and waist-to-height ratio (WHtR). Hazard ratios (HRs) with 95% confidence intervals (CIs) were calculated from Cox regression models and each model fit was assessed using −2log-likelihood.

**Results:**

Every measurement of adiposity was associated with the risk for total stroke and ischemic stroke, but not for hemorrhagic stroke. After adjusting for confounders and intermediates, the HR (comparing the mean of the highest quintile with that of the lowest quintile) for total stroke was 1.34(1.13–1.60) for BMI, 1.26(1.06–1.52) for WC, 1.29(1.08–1.56) for WHpR, and 1.38(1.15–1.66) for WHtR. The HR for ischemic stroke was 1.52(1.24–1.88) for BMI, 1.46(1.17–1.81) for WC, 1.40(1.12–1.74) for WHpR, and 1.62(1.29–2.04) for WHtR. The model fit for each of the indices was similar.

**Conclusions:**

Adiposity increases the total risk of stroke and ischemic stroke, but not of hemorrhagic stroke. No clinically meaningful differences among the associations between BMI, WC, WHpR, and WHtR and stroke incidence were identified in this study.

## Introduction

Stroke is the leading cause of death and long-term disability in China [1], [2], where rising incidence and the impact of stroke have created a serious public health problem [3]. With a rapidly developing economy, obesity in China has increased by 13% in urban areas and 85% in rural regions [4]. The association between obesity and total stroke or ischemic stroke is well recognized. However, the association between obesity and the risk of hemorrhagic stroke remains unclear, especially in Asia. In addition, most epidemiologic investigations have used body mass index (BMI) as the measure of adiposity, while some studies showed that BMI was not an appropriate index to assess the risk of stroke [5], [6]. Several studies have indicated that anthropometric indices of abdominal adiposity, including waist circumference (WC), waist-to-hip ratio (WHpR), waist-to-height ratio (WHtR), are more strongly associated with stroke than BMI [6], [7]. Thus, in this study, we prospectively examined the associations of general adiposity index (BMI) and abdominal adiposity index (WC, WHpR, or WHtR) with risk of stroke subtypes in the Kailuan study.

## Methods

### Study Design and Population

The Kailuan study was a prospective population-based cohort study in the Kailuan community in Tangshan City, which is a large and littoral modern city located in the central area of the circulating Bohai Sea Gulf region. This study was designed to investigate risk factors for chronic diseases (such as stroke, myocardial infarction, cancers, etc.). The Kailuan community is a functional and comprehensive community owned and managed by the Kailuan Group. There are 11 hospitals responsible for the healthcare of the community. All 155,418 residents in the Kailuan community have routine medical examinations, which include physical examination, and routine blood, urine, and biochemical tests every two years without charge. From June 2006 to October 2007, the residents from the Kailuan community who visited the 11 hospitals for routine medical examination and who met the following criteria were invited to participate in the Kailuan study: (1) aged 18 years or older; (2) provided informed consent. In total, 101,510 (65.31%) residents (81,110 men and 20,400 women, aged 18–98 years old) were enrolled in the Kailuan study. The exclusion criteria for the current study were: (1) a past history of myocardial infarction or stroke (n = 3,669); (2) lack of data about measures of adiposity at baseline (n = 3,097). Therefore, 94,744 participants met the selection criteria in this current study. All participants underwent questionnaire assessment, clinical examination, and laboratory assessment. Standard protocols were employed, as described previously [8]. All the doctors and nurses had rigorous unified training before they measured the anthropometric indices.

### Ethics Statement

Our study was approved by the Ethics Committee of both Kailuan General Hospital and Beijing Tiantan Hospital, in compliance with the Declaration of Helsinki. All participants or their legal representatives (for participants with dementia or illiteracy) signed informed consent forms (ICFs).

### Assessment of Anthropometric Indices of Adiposity

All participants were shoeless and wearing scrubs when they were measured. Height was measured to the nearest 0.1 cm using a tape measure, and weight was measured to the nearest 0.1 kg using a calibrated platform scale. WC was measured at the level of the umbilicus, and hip circumference was measured at the point of maximal protrusion of the gluteal muscles; and both were measured to the nearest 0.1 cm. The above anthropometric indices were assessed once. BMI was calculated as body weight (kg) divided by the square of height (m^2^), WHpR was calculated as WC divided by hip circumference, and WHtR was calculated as WC divided by height.

### Assessment of Potential Covariates

Information on demographic variables (e.g., age, sex, household income, education level, and marital status) was collected via questionnaires, which were administered in person by well-trained research doctors and nurses. The average monthly income of each family member was reported as “<¥600”, “¥600–799”, “¥800–999”, or “≥¥1,000”. The education level was categorized as “elementary or below”, “middle school”, “high school”, or “college or above”. The marital status was categorized as “single”, “married”, “divorced”, or “widowed”.

The data on smoking status, drinking status, and physical activity were also collected via questionnaires. Both smoking status and drinking status were classified as “never”, “former”, or “current” according to self-reported information. Physical activity was evaluated based on the responses to questions regarding the type and frequency of physical activity at work and during leisure time. Physical activity was classified as “>4 times per week and >20 minutes at a time”, “<80 minutes per week”, or “none”.

Blood pressure was measured on the left arm to the nearest 2 mm Hg using a mercury sphygmomanometer with a cuff of appropriate size. Two readings of systolic blood pressure (SBP) and diastolic blood pressure (DBP) were taken at 5-minute intervals after participants had rested in a chair for ≥5 minutes. The average of the two readings was used for the current data analyses. If the difference between the two measurements exceeded 5 mm Hg, an additional reading was taken, and the average of the three readings was used. Hypertension was defined as having a history of hypertension, a SBP≥140 mmHg, a DPB ≥90 mmHg, or using antihypertensive medications.

Blood samples were collected from the antecubital vein in the morning after an overnight fast and transfused into vacuum tubes containing EDTA. Fasting blood glucose was measured with the hexokinase/glucose-6-phosphate dehydrogenase method, and cholesterol was measured enzymatically (inter-assay coefficient of variation: <10%; Mind Bioengineering Co. Ltd, Shanghai, China). All blood variables were measured using an auto-analyzer (Hitachi 747; Hitachi, Tokyo, Japan) at the central laboratory of the Kailuan General Hospital. Diabetes mellitus was diagnosed if the subject has a past history of diabetes mellitus, was currently undergoing treatment with insulin or oral hypoglycemic agents, or the fasting blood glucose level was ≥126 mg/dL. Hyperlipidemia was defined as having a history of hyperlipidemia, total blood cholesterol levels ≥220 mg/dL, triglyceride levels ≥150 mg/dL, or using antihyperlipidemic medications.

### Follow-up and Outcome Ascertainment

The participants were followed up by face-to-face interviews at every two-year routine medical examination until December 31, 2010 or to the event of interest or death. The follow-ups were performed by hospital physicians, research physicians, and research nurses who were blinded to the baseline data. The outcome information for the participants without face-to-face follow-up was obtained by referring to death certificates from provincial vital statistics offices, discharge summaries from the 11 hospitals, and medical records from medical insurance.

The primary outcome was the first occurrence of stroke; either the first nonfatal stroke event or a stroke death. A nonfatal stroke was defined as a focal neurological deficit of sudden onset and vascular mechanism that lasted >24 hours. Stroke was diagnosed according to World Health Organization (WHO) criteria [9] combined with brain computed tomography (CT) or magnetic resonance (MR) confirmation, and classified into three main types: brain infarction, intracerebral hemorrhage, or subarachnoid hemorrhage. The criteria were consistent across all participating hospitals.

All stroke records were reviewed by two independent stroke specialists. If instances of disagreement in a single case, the final evaluation was made by the event adjudication committee. All the stroke outcomes were validated by the Data Safety Monitoring Board and Arbitration Committee for Clinical Outcomes. Due to the low incidence and different pathogenesis, we excluded subarachnoid hemorrhage from the following analyses.

### Statistical Analyses

We classified participants into four groups based on WHO BMI categories [10]: underweight (<18.50 kg/m^2^), normal weight (18.50–24.99 kg/m^2^), overweight (25.00–29.99 kg/m^2^), and obesity (≥30.00 kg/m^2^). BMI, WC, WHpR, and WHtR were also classified into quintiles for ease of comparison of the different anthropometric indices. Continuous variables were described by means and categorical variables were described by percentages.

Since no interactions of sex were observed (*P*>0.05) for adiposity indices quintiles, analyses were not done by gender. For each index, we used a Cox proportional hazards model to compute hazard ratios and their 95% confidence intervals (CIs) for total stroke, ischemic stroke, and hemorrhagic stroke by treating the normal weight group or the lowest quintile (the lowest fifth of the data) as the reference category. Person-years of follow-up were calculated as the time from baseline assessment to development of the first endpoint of interest, censoring, or the end of follow-up (December 31, 2010), whichever occurred first.

We created three multivariate-adjusted models. Model 1 was stratified by hospitals and adjusted for age and sex. Anthropometric indices were evaluated by: (1) comparing the strengths of the associations with stroke, and (2) comparing model fit by −2log-likelihood (the model with a smaller value of −2log-likelihood is preferable). In addition to adjusting for the confounders in Model 1, Model 2 also adjusted for the following confounders: average monthly income of each family member, education level, marital status, smoking status, drinking status and physical activity. In addition to the confounders that were adjusted for in Model 2, Model 3 adjusted for the intermediate variables: history of hypertension, diabetes mellitus, and dyslipidemia. Finally, for each model, a trend test was performed after the median value of each quintile was entered into the model and treated as a continuous variable.

Two-sided *P*-values were reported for all analyses. A *P*-value <0.05 was considered to be statistically significant. All statistical analyses were performed by SAS software, version 9.2 (SAS Institute Inc., Cary, NC, USA).

## Results

The mean values of BMI, WC, WHpR, and WHtR were 25.02 kg/m^2^, 86.89 cm, 0.89, and 0.52, respectively at baseline. Selected characteristics of the study population according to the WHO BMI categories and quintiles of BMI are shown in Tables 1 and 2, respectively. Participants with higher BMI had higher values for WC, WHpR, and WHtR, and more frequently reported a history of hypertension, diabetes mellitus, or dyslipidemia, as well as more physical activity. In addition, these subjects were less likely to be current smokers. During an average follow-up of 4 years, 1,141 participants had ischemic stroke and 406 had hemorrhagic stroke among 94,744 participants (20.59% women and 79.41% men). After adjusting for age and gender, the stroke incidence was 42 per 10,000 person-years.

**Table 1:** Selected participant characteristics according to body mass index (BMI).

Characteristics		BMI, kg/m^2^		
	<18.50	18.50–24.99	25.00–29.99	≥30.00
Sample size	1,726	47,504	38,035	7,479
Mean age, years	50.58	50.96	51.70	50.55
Mean waist circumference, cm	73.84	82.34	90.85	98.66
Mean waist-to-hip ratio	0.85	0.88	0.91	0.92
Mean waist-to-height ratio	0.44	0.49	0.54	0.59
Marital status (married), %	86.04	93.20	94.32	94.00
High-school graduate, %	28.22	20.72	18.48	18.42
Average income of each family member ≥800¥, %	13.85	13.61	14.37	13.53
Physical activity >4 times/week, %	13.09	14.45	15.16	15.19
Past smoker,%	4.00	4.42	6.05	5.90
Current smoker,%	33.02	34.16	33.58	31.02
Past/current alcohol drinker, %	34.47	39.40	41.31	38.78
History of hypertension,%	19.87	34.25	51.12	65.06
History of diabetes mellitus,%	4.35	6.61	11.18	14.16
History of dyslipidemia,%	19.29	27.75	41.34	50.30

**Table 2:** Selected participant characteristics according to quintiles of body mass index (BMI).

	BMI Quintile*				
	Q1	Q2	Q3	Q4	Q5
Sample size	18,923	18,930	18,869	19,096	18,926
Mean age, years	50.06	51.34	51.79	51.82	51.08
Mean waist circumference, cm	78.32	83.24	86.82	90.30	95.72
Mean waist-to-hip ratio	0.86	0.88	0.90	0.91	0.92
Mean waist-to-height ratio	0.47	0.50	0.52	0.54	0.57
Marital status (married), %	91.59	93.66	93.96	94.62	94.08
High-school graduate, %	23.75	19.71	18.82	17.93	18.69
Average income of each family ≥800 ¥, %	13.76	13.67	13.62	14.25	14.27
Physical activity >4 times/week, %	13.66	15.05	14.86	14.92	15.33
Past smoker,%	3.56	4.82	5.33	6.09	6.11
Current smoker,%	34.05	34.03	34.45	33.58	32.17
Past/current alcohol drinker, %	37.39	40.27	41.07	41.26	40.13
History of hypertension,%	25.84	36.31	43.42	50.28	60.05
History of diabetes mellitus,%	4.71	7.09	8.94	11.23	13.02
History of dyslipidemia,%	22.28	28.72	35.21	40.51	47.40

Q1, the lowest fifth of the data (1–20%); Q2,the second lowest fifth (21–40%); Q3,the middle fifth (41–60%); Q4, the second highest fifth (61–80%); Q5,the highest fifth (81–100%).

* Cut-points of quintile of BMI (Kg/m^2^): 22.05, 24.00, 25.71, 27.74.

Table 3 summarizes the HRs for stroke according to the WHO categories of BMI, model fit results, and *P*-trend values. There was no significant association between BMI and stroke in the underweight group (<18.50 kg/m^2^) compared with the normal weight group (18.50–24.99 kg/m^2^). Comparisons of the overweight (25.00–29.99 kg/m^2^), and obesity (≥30.00 kg/m^2^) with the normal weight (18.50–24.99 kg/m^2^) groups showed a significant positive correlation between BMI and total and ischemic stroke, although no significant association was observed between BMI and hemorrhagic stroke. The *P*-values for trend were significant for total and ischemic stroke, but not for hemorrhagic stroke.

**Table 3:** Hazard ratios (95% CI) for stroke according to WHO categories of BMI.

	BMI(kg/m^2^)				−2log L	P for trend
	<18.50	18.50–24.99	25.00–29.99	≥30.00		
No. of subjects	1726	47504	38035	7479		
Total stroke						
Cases	15	670	709	153		
Model 1†	0.49(0.30–0.82)	1	1.32(1.19–1.47)	1.55(1.30–1.84)	28130	<.01
Model 2‡	0.47(0.28–0.79)	1	1.34(1.20–1.49)	1.56(1.31–1.87)	28051	<.01
Model 3§	0.56(0.33–0.93)	1	1.13(1.02–1.26)	1.17(0.98–1.40)	27763	0.01
Ischemic stroke						
Cases	11	476	539	115		
Model 1†	0.49(0.27–0.90)	1	1.42(1.26–1.61)	1.64(1.34–2.01)	20556	<.01
Model 2‡	0.47(0.259–0.86)	1	1.44(1.27–1.63)	1.67(1.36–2.05)	20471	<.01
Model 3§	0.56(0.30–1.01)	1	1.21(1.07–1.37)	1.24(1.00–1.53)	20246	<.01
Hemorrhagic stroke						
Cases	4	194	170	38		
Model 1†	0.49(0.18–1.32)	1	1.09(0.89–1.34)	1.32(0.93–1.87)	7573	0.15
Model 2‡	0.48(0.18–1.28)	1	1.09(0.89–1.34)	1.31(0.92–1.86)	7547	0.16
Model 3§	0.56(0.21–1.52)	1	0.94(0.76–1.16)	1.00(0.70–1.42)	7466	0.68

CI, confidence interval; WHO, World Health Organization; BMI, body mass index;

† Model 1 was stratified by hospitals, and adjusted for age and sex.

‡ Model 2 was stratified by hospitals, and adjusted for age, sex, average monthly income of each family member, education level, marital status, smoking status, drinking status, and physical activity.

§ Model 3 was stratified by hospitals, and adjusted for age, sex, average monthly income of each family member, education level, marital status, smoking status, drinking status, physical activity, history of hypertension, diabetes mellitus, and dyslipidemia.

Table 4 summarizes the HRs for total stroke according to quintiles of various adiposity indices, model fit results, and *P*-trend values in the models. In all the multivariate-adjusted analyses, BMI, WC, WHpR, and WHtR were correlated linearly with risk for total stroke (all *P*-values for trend <0.01). Comparing the highest with the lowest quintile of these measurements, the adjusted HR (95% CI) for total stroke in Model 2 was 1.88(1.59–2.22) for BMI, 1.71(1.43–2.04) for WC, 1.52(1.27–1.83) for WHpR, and 1.84(1.53–2.21) for WHtR. After adjusting more intermediate variables in Model 3 (i.e., histories of hypertension, diabetes mellitus, and dyslipidemia), this association was attenuated, but remained significant. The adjusted HR (95% CI) for total stroke in Model 3 was 1.34(1.13–1.60) for BMI, 1.26(1.06–1.52) for WC, 1.29(1.08–1.56) for WHpR, and 1.38(1.15–1.66) for WHtR. In addition, the model fit in Model 3, as assessed by −2log-likelihood, was 27,759 for BMI, 27,761 for WC, 27,763 for WHpR, and 27,750 for WHtR. Overall, the model with WHtR had the best model fit.

**Table 4:** Hazard ratios (95% CI) for total stroke according to quintiles of BMI, WC, WHpR, and WHtR.

	Quintile*					−2log L	P for trend
	Q1	Q2	Q3	Q4	Q5		
**BMI**							
No. of subjects(cases)	18,923(219)	18,930(261)	18,869(332)	19,096(350)	18,926(385)		
Model 1†	1	1.19(1.00–1.43)	1.52(1.28–1.80)	1.60(1.35–1.89)	1.83(1.55–2.17)	28117	<.01
Model 2‡	1	1.21(1.01–1.44)	1.54(1.30–1.83)	1.62(1.37–1.92)	1.88(1.59–2.22)	28037	<.01
Model 3§	1	1.08(0.90–1.30)	1.29(1.09–1.54)	1.26(1.06–1.50)	1.34(1.13–1.60)	27759	<.01
**WC**							
No. of subjects(cases)	19,797(175)	18,334(229)	20,035(317)	16,317(346)	20,261(480)		
Model 1†	1	1.17(0.96–1.43)	1.40(1.16–1.68)	1.68(1.40–2.02)	1.70(1.43–2.03)	28130	<.01
Model 2‡	1	1.17(0.96–1.43)	1.40(1.16–1.68)	1.70(1.41–2.04)	1.71(1.43–2.04)	28053	<.01
Model 3§	1	1.06(0.87–1.29)	1.18(0.98–1.42)	1.35(1.12–1.62)	1.26(1.06–1.52)	27761	<.01
**WHpR**							
No. of subjects(cases)	18,956(178)	18,863(270)	18,971(333)	19,198(382)	18,756(384)		
Model 1†	1	1.27(1.05–1.54)	1.51(1.25–1.82)	1.57(1.31–1.88)	1.55(1.29–1.87)	28151	<.01
Model 2‡	1	1.27(1.05–1.53)	1.50(1.24–1.80)	1.56(1.30–1.87)	1.52(1.27–1.83)	28078	<.01
Model 3§	1	1.18(0.98–1.43)	1.33(1.10–1.60)	1.35(1.12–1.62)	1.29(1.08–1.56)	27763	<.01
**WHtR**							
No. of subjects(cases)	19,048(162)	18,783(218)	19,023(277)	18,975(406)	18,915(484)		
Model 1†	1	1.14(0.93–1.40)	1.32(1.09–1.60)	1.73(1.44–2.08)	1.83(1.52–2.19)	28111	<.01
Model 2‡	1	1.14(0.93–1.40)	1.33(1.10–1.62)	1.75(1.46–2.10)	1.84(1.53–2.21)	28035	<.01
Model 3§	1	1.04(0.85–1.28)	1.14(0.94–1.39)	1.41(1.17–1.70)	1.38(1.15–1.66)	27750	<.01

CI, confidence interval; BMI, body mass index; WC, waist circumference; WHpR, waist-to-hip ratio; WHtR, waist-to-height; −2log L, −2log-likelihood; Q1, the lowest fifth of the data (1–20%); Q2,the second lowest fifth (21–40%); Q3,the middle fifth (41–60%);Q4, the second highest fifth (61–80%);Q5,the highest fifth (81–100%).

* Cut-points of quintiles: BMI (Kg/m^2^) 22.05, 24.00, 25.71, 27.74; WC (cm) 79.00, 84.00, 89.00, 95.00; WHpR 0.84, 0.88, 0.91, 0.94; WHtR 0.47, 0.50, 0.53, 0.57.

† Model 1 was stratified by hospitals, and adjusted for age and sex.

‡ Model 2 was stratified by hospitals, and adjusted for age, sex, average monthly income of each family member, education level, marital status, smoking status, drinking status, and physical activity.

§ Model 3 was stratified by hospitals, and adjusted for age, sex, average monthly income of each family member, education level, marital status, smoking status, drinking status, physical activity, history of hypertension, diabetes mellitus, and dyslipidemia.

As shown in Table 5, the adjusted HR (95% CI) for ischemic stroke, comparing the highest with the lowest quintile of these measurements, were 1.52(1.24–1.88) for BMI, 1.46(1.17–1.81) for WC, 1.40(1.12–1.74) for WHpR, and 1.62(1.29–2.04) for WHtR, after adjustment for all the potential confounders and intermediate variables in Model 3 (Table 5). Ischemic stroke was positively and strongly associated with all obesity measurements.

**Table 5:** Hazard ratios (95% CI) for ischemic stroke according to quintiles of BMI, WC, WHpR, and WHtR.

	Quintile*					−2log L	P for trend
	Q1	Q2	Q3	Q4	Q5		
**BMI**							
No. of subjects(cases)	18,923(143)	18,930(189)	18,869(259)	19,096(267)	18,926(283)		
Model 1†	1	1.32(1.06–1.64)	1.82(1.48–2.23)	1.87(1.53–2.30)	2.07(1.69–2.54)	20537	<.01
Model 2‡	1	1.35(1.08–1.67)	1.86(1.51–2.28)	1.92(1.57–2.36)	2.14(1.75–2.63)	20452	<.01
Model 3§	1	1.20(0.97–1.50)	1.55(1.26–1.91)	1.48(1.21–1.83)	1.52(1.24–1.88)	20236	<.01
**WC**							
No. of subjects(cases)	19,797(116)	18,334(164)	20,035(233)	16,317(255)	20,261(373)		
Model 1†	1	1.27(1.00–1.62)	1.55(1.24–1.94)	1.85(1.48–2.31)	1.98(1.60–2.45)	20552	<.01
Model 2‡	1	1.27(1.00–1.62)	1.56(1.25–1.95)	1.87(1.50–2.34)	2.00(1.62–2.48)	20469	<.01
Model 3§	1	1.14(0.90–1.45)	1.31(1.04–1.64)	1.47(1.18–1.84)	1.46(1.17–1.81)	20244	<.01
**WHpR**							
No. of subjects(cases)	18,956(123)	18,863(204)	18,971(249)	19,198(281)	18,756(284)		
Model 1†	1	1.39(1.11–1.74)	1.65(1.32–2.06)	1.68(1.36–2.09)	1.70(1.36–2.11)	20577.086	<.01
Model 2‡	1	1.39(1.11–1.74)	1.64(1.32–2.05)	1.68(1.35–2.08)	1.68(1.35–2.09)	20498.291	<.01
Model 3§	1	1.30(1.03–1.62)	1.45(1.160–1.80)	1.44(1.16–1.79)	1.40(1.12–1.74)	20248	<.01
**WHtR**							
No. of subjects(cases)	19,048(104)	18,783(157)	19,023(205)	18,975(303)	18,915(372)		
Model 1†	1	1.28(1.00–1.64)	1.52(1.20–1.93)	1.98(1.58–2.48)	2.16(1.73–2.70)	20536.417	<.01
Model 2‡	1	1.29(1.00–1.65)	1.54(1.22–1.96)	2.01(1.61–2.52)	2.20(1.76–2.75)	20453	<.01
Model 3§	1	1.17(0.91–1.50)	1.31(1.03–1.66)	1.61(1.28–2.02)	1.62(1.29–2.04)	20233	<.01

CI, confidence interval; BMI, body mass index; WC, waist circumference; WHpR, waist-to-hip ratio; WHtR, waist-to-height; −2log L, −2log-likelihood; Q1, the lowest fifth of the data (1–20%); Q2,the second lowest fifth (21–40%); Q3,the middle fifth (41–60%);Q4, the second highest fifth (61–80%); Q5, the highest fifth (81–100%).

* Cut-points of quintiles: BMI (Kg/m^2^) 22.05, 24.00, 25.71, 27.74; WC (cm) 79.00, 84.00, 89.00, 95.00; WHpR 0.84, 0.88, 0.91, 0.94; WHtR 0.47, 0.50, 0.53, 0.57.

† Model 1 was stratified by hospitals, and adjusted for age and sex.

‡ Model 2 was stratified by hospitals, and adjusted for age, sex, average monthly income of each family member, education level, marital status, smoking status, drinking status, and physical activity.

§ Model 3 was stratified by hospitals, and adjusted for age, sex, average monthly income of each family member, education level, marital status, smoking status, drinking status, physical activity, history of hypertension, diabetes mellitus, and dyslipidemia.

The association between quintiles of various measures of adiposity and hemorrhagic stroke is reported in Table 6. Comparison of the highest with the lowest quintile of these measurements showed a significant positive correlation between BMI and hemorrhagic stroke in Models 1 and 2, but no significant association was observed in Model 3. In contrast, there was no significant correlation of WC, WHtR, and WHpR with hemorrhagic stroke in any of the models. The *P*-values for trend were significant for BMI and WHtR, but not for WC and WHpR in Model 1 and Model 2 and not for any indices of adiposity in Model 3. Furthermore, subgroup analysis of the relationship between adiposity indices and risk of stroke in the participants ≥40 years showed similar results to the main group (Table 7).

**Table 6:** Hazard ratios (95% CI) for hemorrhagic stroke according to quintiles of BMI, WC, WHpR, and WHtR.

	Quintile*					−2log L	P for trend
	Q1	Q2	Q3	Q4	Q5		
**BMI**							
No. of subjects(cases)	18,923(76)	18,930(72)	18,869(73)	19,096(83)	18,926(102)		
Model 1†	1	0.95(0.69–1.31)	0.96(0.69–1.32)	1.08(0.79–1.47)	1.39(1.03–1.87)	7570	0.02
Model 2‡	1	0.95(0.69–1.32)	0.96(0.69–1.32)	1.08(0.78–1.47)	1.39(1.03–1.87)	7544	0.02
Model 3§	1	0.86(0.62–1.19)	0.81(0.59–1.13)	0.86(0.63–1.19)	1.02(0.75–1.39)	7465	0.80
**WC**							
No. of subjects(cases)	19,797(59)	18,334(65)	20,035(84)	16,317(91)	20,261(107)		
Model 1†	1	0.98(0.69–1.40)	1.09(0.78–1.53)	1.36(0.97–1.89)	1.16(0.84–1.61)	7574	0.12
Model 2‡	1	0.98(0.69–1.40)	1.09(0.78–1.52)	1.35(0.97–1.88)	1.15(0.83–1.59)	7548	0.13
Model 3§	1	0.89(0.62–1.27)	0.94(0.67–1.32)	1.11(0.79–1.55)	0.89(0.64–1.25)	7465	0.91
**WHpR**							
No. of subjects(cases)	18,956(55)	18,863(66)	18,971(84)	19,198(101)	18,756(100)		
Model 1†	1	1.00(0.70–1.43)	1.18(0.84–1.68)	1.30(0.92–1.82)	1.22(0.86–1.72)	7575	0.10
Model 2‡	1	1.00(0.69–1.43)	1.18(0.83–1.67)	1.28(0.91–1.80)	1.18(0.84–1.67)	7549	0.14
Model 3§	1	0.94(0.65–1.34)	1.06(0.75–1.51)	1.14(0.81–1.60)	1.06(0.75–1.49)	7466	0.43
**WHtR**							
No. of subjects(cases)	19,048(58)	18,783(61)	19,023(72)	18,975(103)	18,915(112)		
Model 1†	1	0.89(0.62–1.28)	0.97(0.68–1.37)	1.28(0.92–1.78)	1.22(0.88–1.70)	7571	0.03
Model 2‡	1	0.89(0.62–1.28)	0.97(0.68–1.37)	1.28(0.92–1.78)	1.21(0.87–1.68)	7545	0.03
Model 3§	1	0.82(0.57–1.18)	0.85(0.60–1.20)	1.07(0.77–1.49)	0.96(0.69–1.34)	7464	0.54

CI, confidence interval; BMI, body mass index; WC, waist circumference; WHpR, waist-to-hip ratio; WHtR, waist-to-height; −2log L, −2log-likelihood; Q1, the lowest fifth of the data (1–20%); Q2,the second lowest fifth (21–40%); Q3,the middle fifth (41–60%);Q4, the second highest fifth (61–80%); Q5, the highest fifth (81–100%).

* Cut-points of quintiles: BMI (Kg/m^2^) 22.05, 24.00, 25.71, 27.74; WC (cm) 79.00, 84.00, 89.00, 95.00; WHpR 0.84, 0.88, 0.91, 0.94; WHtR 0.47, 0.50, 0.53, 0.57.

† Model 1 was stratified by hospitals, and adjusted for age and sex.

‡ Model 2 was stratified by hospitals, and adjusted for age, sex, average monthly income of each family member, education level, marital status, smoking status, drinking status, and physical activity.

§ Model 3 was stratified by hospitals, and adjusted for age, sex, average monthly income of each family member, education level, marital status, smoking status, drinking status, physical activity, history of hypertension, diabetes mellitus, and dyslipidemia.

**Table 7:** Hazard ratios (95% CI) for stroke according to quintiles of BMI, WC, WHpR, and WHtR in participants aged ≥40 years.

	Quintile*					−2log L	P for trend
	Q1	Q2	Q3	Q4	Q5		
**Total stroke**							
BMI	1	1.19(1.00–1.42)	1.30(1.09–1.55)	1.32(1.11–1.57)	1.38(1.16–1.64)	26956	<.01
WC	1	1.07(0.87–1.30)	1.17(0.97–1.41)	1.30(1.08–1.57)	1.28(1.06–1.55)	26960	<.01
WHpR	1	1.11(0.92–1.33)	1.30(1.09–1.55)	1.31(1.10–1.56)	1.26(1.05–1.50)	26959	<.01
WHtR	1	0.98(0.80–1.19)	1.16(0.96–1.40)	1.39(1.17–1.66)	1.29(1.08–1.54)	26947	<.01
**Ischemic stroke**							
BMI	1	1.33(1.07–1.64)	1.56(1.26–1.92)	1.56(1.27–1.92)	1.56(1.26–1.92)	19857	<.01
WC	1	1.18(0.93–1.50)	1.30(1.03–1.63)	1.46(1.17–1.82)	1.47(1.18–1.84)	19866	<.01
WHpR	1	1.13(0.91–1.40)	1.37(1.11–1.68)	1.34(1.09–1.64)	1.29(1.05–1.59)	19870	<.01
WHtR	1	1.08(0.85–1.36)	1.27(1.02–1.58)	1.55(1.26–1.91)	1.48(1.20–1.83)	19855	<.01
**Hemorrhagic stroke**							
BMI	1	0.94(0.68–1.29)	0.81(0.58–1.13)	0.86(0.62–1.20)	1.04(0.76–1.43)	7042	0.87
WC	1	0.84(0.58–1.21)	0.92(0.65–1.30)	0.99(0.70–1.39)	0.92(0.65–1.29)	7044	0.97
WHpR	1	1.05(0.74–1.50)	1.11(0.78–1.59)	1.24(0.88–1.74)	1.15(0.81–1.63)	7043	0.28
WHtR	1	0.78(0.54–1.11)	0.94(0.67–1.32)	1.06(0.77–1.47)	0.92(0.66–1.28)	7041	0.72

CI, confidence interval; BMI, body mass index; WC, waist circumference; WHpR, waist-to-hip ratio; WHtR, waist-to-height; −2log L, −2log-likelihood; Q1, the lowest fifth of the data (1–20%); Q2, the second lowest fifth (21–40%); Q3, the middle fifth (41–60%);Q4, the second highest fifth (61–80%); Q5, the highest fifth (81–100%).

* Cut-points of quintiles: BMI (Kg/m^2^) 22.23, 24.11, 25.78, 27.76; WC(cm) 80.00, 85.00, 90.00, 95.00; WHpR 0.85, 0.88, 0.91, 0.95; WHtR 0.48, 0.51, 0.54, 0.57.

* Multivariate Model was stratified by hospitals, and adjusted for age, sex, average monthly income of each family member, education level, marital status, smoking status, drinking status, physical activity, history of hypertension, diabetes mellitus, and dyslipidemia.

## Discussion

In this large prospective cohort study of 94,744 participants in the Kailuan study, a significant linear association of high levels of BMI, WC, WHpR, and WHtR with increased risk of total stroke and ischemic stroke was observed. This association persisted after controlling for potential confounders and intermediate variables although it was attenuated after adjustment for intermediate variables. This indicated that obesity is involved in the causal pathway leading to the more proximal risk factors for stroke and contribute to stroke risk through those factors. BMI and WHtR showed a significant association with hemorrhagic stroke in Model 1 and Model 2, although there were no associations between all indices of adiposity and risk for hemorrhagic stroke after adjustment for potential confounding factors and intermediate variables in Model 3. These data suggest that both general and abdominal adiposity indices were associated with increased risk of total stroke and ischemic stroke, but not of hemorrhagic stroke. Although obesity was strongly associated with all intermediate variables (hypertension, diabetes mellitus, and dyslipidemia), the association between adiposity indices and the risks of total and ischemic stroke remained statistically significant after adjustment for these factors in our study. This suggests that the increased risk of stroke associated with high values of adiposity indices are not be solely mediated by the development of hypertension, diabetes mellitus, and dyslipidemia.

The significant associations between obesity and risk of total stroke and ischemic stroke are consistent with findings in earlier studies in Asian [11], [12], [13] and Western populations [6], [14], [15], [16], [17].

However, the relationship between obesity and risk of hemorrhagic stroke is still controversial. Some studies [6], [11], [12], [14], [15], [16] have shown that there is no relationship between obesity and the risk of hemorrhagic stroke after adjustment for potential confounding factors and intermediate variables, which is in accordance with our findings. However, others found a positive correlation [13], [17], or an inverse association for this relationship [18].

It has been recognized that BMI does not distinguish subjects with excess adiposity tissue from those with high muscle mass; therefore, this characteristic may incorrectly reflect the risk of stroke for subjects with high muscle mass [19], [20], [21].

We did not find substantial or clinically meaningful differences among the relationships of BMI, WC, WHpR, or WHtR with stroke. Several studies [5], [22], [23], [24] have shown that WHpR, rather than BMI, is a promising predictor of stroke incidence, but others suggested a stronger association between WC [23], [25], [26], [27] or WHtR [28], [29] and stroke incidence. Furthermore, some researchers [30] suggested that there were no differences in the relationships between each index of adiposity and risk of stroke. For example, Zhang et al. found that the estimators of general adiposity (BMI) and abdominal (WC, WHpR, WHtR) adiposity did not differ in their predictive value for stroke in Chinese women [13], which is consistent with our findings.

It is worth noting that some studies found an inverse relationship between body weight and mortality in patients after stroke. In the FOOD trial [31], the investigators reported that undernourished patients had a 2.3-fold higher risk of dying within 6 months compared with patients with normal nutritional status, whereas overweight patients had no increased risk of death. In the Danish National Indicator Project (NIP) including data from 21,884 stroke patients, the highest mortality was observed in the underweight patients and patients with overweight and obesity had a significantly higher survival rate than patients with normal body weight [32]. Additionally, in a recent Greek study conducted in 2,785 stroke patients, an inverse association between body weight and mortality was also reported [33]. It can be speculated that the advantage of higher body weight is due to the overall catabolic imbalance related to the underlying diseases [34].

This study has several strengths, including the prospective design, large sample size in an Asian population, enrollment of women and men, standardized evaluation of directly measured body size, broad assessment of potential confounders, and confirmation of stroke events through reviewing medical records. Compared with other studies in Asian populations, this is the first study to assess the relationship between every anthropometric index of abdominal adiposity and stroke (including total stroke and stroke subtypes) among men and women. Two studies in Japanese populations assessed only the association of BMI and WC with stroke [12], [18]. Another Chinese study assessed the relationship between stroke and all of the anthropometric indices of abdominal adiposity such as BMI, WC, WHpR, WHtR, although only female participants were included, and the investigators did not distinguish between ischemic stroke and hemorrhagic stroke at the end points [13]. However, our study has some limitations. First, the participants in the Kailuan study do not constitute a nationally representative sample and our findings may not be generalized directly to other Chinese populations with different educational and cultural backgrounds. Second, indices of adiposity were measured once at the baseline; therefore, evaluation of changes in body size was not possible. Finally, the number of hemorrhagic stroke events might be still insufficient for the detection of minor associations between adiposity indices and hemorrhagic stroke.

### Conclusions

In summary, our study indicates that both general and abdominal adiposity increase the risk of total stroke and ischemic stroke, but not of hemorrhagic stroke. Our data indicate that there are no clinically meaningful differences among the associations between BMI, WC, WHpR, and WHtR and stroke incidence in this study.
