# Correction: Measures of Adiposity and Risk of Stroke in China: A Result from the Kailuan Study

**DOI:** 10.1371/annotation/082957ac-2ece-4ed6-8cab-e02ad0b60d8c

**Published:** 2013-08-22

**Authors:** Anxin Wang, Jianwei Wu, Yong Zhou, Xiuhua Guo, Yanxia Luo, Shouling Wu, Xingquan Zhao

The first two authors, Anxin Wang and Jianwei Wu, should be noted as contributing equally to this work. 

